# Eight and a half syndrome: a rare presentation of a brainstem infarction

**DOI:** 10.1093/omcr/omac089

**Published:** 2022-08-18

**Authors:** Georgios Pilianidis, Georgios Gogos, Christina Tontikidou, Athanasios Christanas, Nikoletta Kosmidou, Iakovos Avramidis

**Affiliations:** Internal Medicine Department, G Papanikolaou Hospital, Thessaloniki, Makedonia Thraki 57010, Greece; Internal Medicine Department, G Papanikolaou Hospital, Thessaloniki, Makedonia Thraki 57010, Greece; Internal Medicine Department, G Papanikolaou Hospital, Thessaloniki, Makedonia Thraki 57010, Greece; Radiology Department, G Papanikolaou Hospital, Thessaloniki, Makedonia Thraki 57010, Greece; Internal Medicine Department, G Papanikolaou Hospital, Thessaloniki, Makedonia Thraki 57010, Greece; Internal Medicine Department, G Papanikolaou Hospital, Thessaloniki, Makedonia Thraki 57010, Greece

Α 75-year-old man known to have arterial hypertension presented with a 2-hour history of dysarthria, diplopia, left facial weakness and abnormal sensation on the left side of his face.

Neurological examination revealed left lower motor neuron facial nerve palsy. Eye examination revealed total left eye horizontal gaze paresis, limitation of right eye adduction with preservation of abduction of the right eye, which evoked a right lateral nystagmus. Vertical eye movements were preserved.

Our patient was diagnosed with eight and a half syndrome characterized by the combination of ipsilateral lower motor neuron VIIth nerve palsy, internuclear ophthalmoplegia and ipsilateral gaze paralysis [1]. The syndrome causes complete ipsilateral seventh lower motor neuron with horizontal gaze paresis alongside partial gaze paresis of the opposite eye (Fig. 1) [2].

His brain magnetic resonance imaging revealed a left pontine infarct involving the para pontine reticular formation, the medial longitudinal fasciculus and the VIIth nerve nucleus.

This syndrome is caused by a lesion that affects the ipsilateral paramedian pontine reticular formation or the abducens nucleus and the ipsilateral medial longitudinal fasciculus [3]. When this lesion affects the fascicle of the ipsilateral facial nerve in the area of the facial colliculus as it wraps around the abducens nucleus, it produces a lower motor neuron pattern of ipsilateral facial weakness [4].

**Figure 1:**
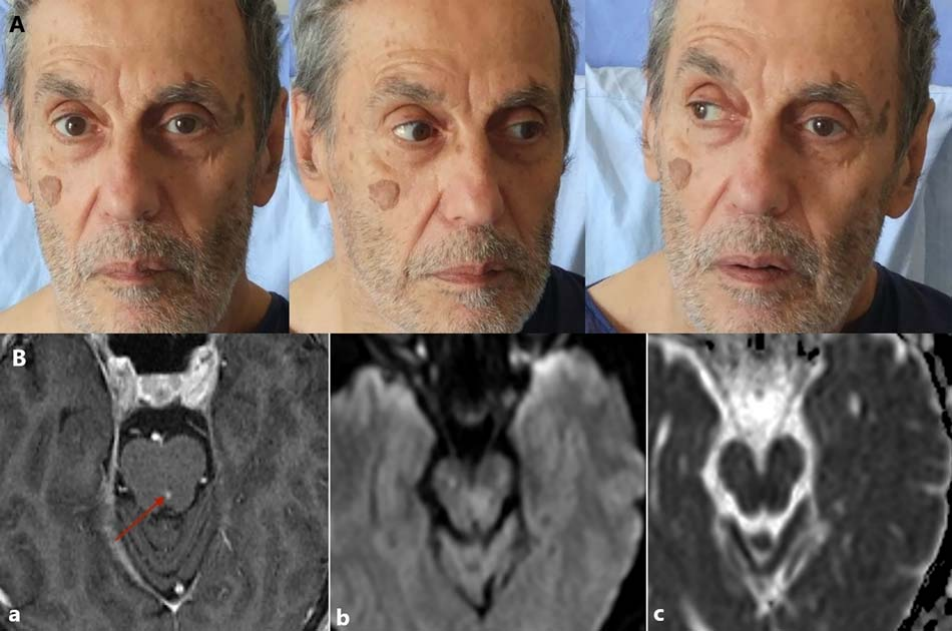
(A) Left VIth and VIIth nerve palsy, internuclear ophthalmoplegia and ipsilateral gaze paresis and partial right horizontal gaze paresis. (B) High signal lesion in midline of pons-left part of midbrain (a). T1 weighted with gadolinium contrast image (b). Restricted diffusion on Diffusion weighted image at the level of the pontine tegmentum (c). Restricted diffusion evident on apparent diffusion coefficient at the same area in the pons.

Commonest causes of eight and a half syndrome include ischemic strokes, demyelinating lesions, tumors and arteriovenous malformations [5].

Recognizing the clinical signs of this syndrome allows the clinician to localize precisely the extent and distribution of the lesion to the left pontine tegmentum, which is supplied by the anterior inferior cerebellar artery or paramedian pontine perforators of the basilar artery, and predict the functional outcome [6].

## CONFLICT OF INTEREST STATEMENT

No competing interests were disclosed.

## FUNDING

There were no sources of funding.

## ETHICAL APPROVAL

Not applicable.

## CONSENT

Written informed consent was obtained from the patient for publication of this case report and any accompanying images. A copy of the written consent is available for review by the Editor-in-Chief of this journal.

## GUARANTOR

Dr Georgios Pilianidis, MD, MSc Infectious Diseases.
